# Investigation and management of the unknown primary with metastatic neck disease: United Kingdom National Multidisciplinary Guidelines

**DOI:** 10.1017/S0022215116000591

**Published:** 2016-05

**Authors:** K Mackenzie, M Watson, P Jankowska, S Bhide, R Simo

**Affiliations:** 1 University of Strathclyde, Glasgow, UK; 2 ENT Department, Doncaster Royal Infirmary, Doncaster, UK; 3 Department of Oncology, Beacon Centre, Taunton and Somerset NHS Foundation Trust, Taunton, UK; 4 Head and Neck Unit, The Royal Marsden NHS Foundation Trust, London, UK; 5 Department of Otolaryngology – Head and Neck Surgery, Guy's and St Thomas’ Hospital NHS Foundation Trust, Department of Otolaryngology – Head and Neck Surgery, Guy's, King's and St Thomas’ Medical and Dental School, London, UK

## Abstract

This is the official guideline endorsed by the specialty associations involved in the care of head and neck cancer patients in the UK. It discusses the evidence base pertaining to the management of metastatic neck disease in the setting of an unknown primary and provides recommendations on the work up and management for this group of patients receiving cancer care.

**Recommendations:**

• All patients presenting with confirmed cervical lymph node metastatic squamous cell carcinoma and no apparent primary site should undergo:
  ○ Positron emission tomography-computed tomography whole-body scan. (R)
  ○ Panendoscopy and directed biopsies. (R)
  ○ Bilateral tonsillectomy. (R)
• Tongue base mucosectomy can be offered if facilities and expertise exists. (G)
• Concomitant chemotherapy with radiation should be considered in patients with an unknown primary. (R)
• Concomitant chemotherapy with radiation should be offered to suitable patients in the post-operative setting, where indicated. (R)
• Neo-adjuvant chemotherapy can be used in gross ‘unresectable’ disease. (R)
• Patients should be followed up at least two months in the first two years and three to six months in the subsequent years. (G)
• Patients should be followed up to a minimum of five years with a prolonged follow up for selected patients. (G)
• Positron emission tomography–computed tomography scan at three to four months after treatment is a useful follow-up strategy for patients treated by chemoradiation therapy. (R)

## Introduction

An unknown primary is defined as a squamous cell carcinoma (SCC) presenting in a lymph node or nodes in the neck with no primary index site in the head and neck having been identified. These patients are best assessed comprehensively through a dedicated neck lump clinic. As part of this assessment the lymph node should be sampled and in general it is recognised that this is best achieved by ultrasound-guided fine needle aspiration (FNA) cytology and/or core biopsy under ultrasound guidance. The receipt of a cytological or histological report confirming SCC initiates the need for further investigation.

## Clinical presentation

Neck lumps presenting with no discernible primaries can be solid or cystic lesions, which can be solitary or multiple lumps. The lumps are usually located in level 2, followed by level 3, with bilateral involvement and other symptoms (i.e. pain and dysphagia) reported in less than 10 per cent. The clinical N stage at presentation is usually N2a, N2b and N2c.^1^ The presence of cystic malignant metastases in level 2 is often considered to be a hallmark of human papilloma virus (HPV)-related squamous carcinoma, usually with subclinical primaries in the oropharynx.^1^ The first echelon lymph node or nodes, which are involved in SCC can act as an indicator for the potential origin of the index primary are shown in Table I.

**Table I:** First echelon lymph nodes for various primary sites

Level 1	Oral cavity, oropharynx
Level 2	Oral cavity, oropharynx, larynx, nose, hypopharynx, parotid, nasopharynx
Level 3	Oral cavity, oropharynx, larynx, hypopharynx, thyroid, nasopharynx
Level 4	Larynx, thyroid, hypopharynx, oesophagus
Level 5	Nasopharynx, hypopharynx, thyroid, oropharynx
Level 6	Thyroid, larynx, hypopharynx, cervical oesophagus

It should be also noted that patients presenting with supraclavicular lymphadenopathy may represent a different clinical entity,^2^ due to the potential for association with infraclavicular neoplasms, such as lung cancer.

## Assessment and staging

Clinical examination of the nose, post-nasal space, oral cavity, oropharynx, larynx and hypopharynx, including palpation of the oral cavity and tongue base should be carried out under direct vision and using rigid and flexible endoscopes as appropriate. The skin and scalp of the head and neck region should be examined to ensure that there are no significant cutaneous lesions. If there is an obvious lesion, or high suspicion of a lesion, then further management in the form of imaging and panendoscopy of that sub-site should be carried out. If there is no obvious or highly suspicious lesion on out-patient assessment, then the patient should be regarded as having an unknown primary and should be evaluated further, this clinical entity being known as a ‘clinical’ unknown primary. To try to determine the site of the primary the following investigations and findings should be collated.

### Pathology of lymph nodes

The advantage of a core biopsy over FNA cytology is that a clearer histological picture can be determined.^3^ Although this is generally used to differentiate between squamous, thyroid, salivary, breast or bronchial origins, it may be possible from the cell architecture to suggest the potential origin of the index primary. Even though immuno-histochemical techniques may not be able to suggest the tumour origin they may, however, potentially exclude sites, e.g. by the use of lung or thyroid markers. More specific investigations such as identification of Epstein-Barr virus (EBV) may correlate highly with a nasopharyngeal site.

Human papilloma virus is a significant aetiological factor in oropharyngeal cancer and so the identification of HPV 16 and 18 in a lymph node sample would be strongly suggestive of an oropharyngeal origin.^1^^,^^4^ P16 positivity is highly predictive of HPV overexpression and may be used as a surrogate marker to indicate the HPV status.

### Cross-sectional imaging

All patients should have computed tomography (CT) imaging from skull base to diaphragm as part of the assessment of a newly diagnosed SCC of the head and neck.^1^ In the clinical scenario of an unknown primary, it would be appropriate to undertake this as it would assess and confirm the extent of the lymphadenopathy and whether there is a second primary or metastasis in the lung. Computed tomography imaging may show evidence of a potential index primary site, although in general, it is infrequently of significant value in diagnosing low-volume tumours in the head and neck. If the disease presents in a level 2/3 lymph node magnetic resonance imaging (MRI) of the oropharynx, and in particular the tongue base, tonsil and tonsil lingual angle, should be carried out. It could be argued that all unknown primary patients should have an MRI of the neck up to skull base. It should be borne in mind, however, that positron emission tomography–computed tomography (PET-CT) may be carried out as the first-line investigation of these patients in which case ‘plain’ CT should not be carried out.

### Positron emission tomography–computed tomography fusion scan

Positron emission tomography-computed tomography scanning is the recognised investigation of choice in the assessment of the unknown primary and has been shown to be superior to CT scanning alone. Recent meta-analysis reported an identification rate of 44 per cent, a sensitivity of 97 per cent and a specificity of 68 per cent.^5^^,^^6^ The evidence in support of this modality is level 3 and is based on observational series. Within this assessment it should be noted that there is a significant false-positive identification rate associated with PET–CT scan. Despite these limitations, PET–CT has now been confirmed as not only the imaging modality of choice in the investigation of an unknown primary, but is now also regarded as the current standard of care.^1^

### Panendoscopy

Following each of the clinical and radiological assessments it is necessary to carry out panendoscopy of the upper aerodigestive tract under general anaesthesia.

The timing of this should be following the completion of all of the imaging as any instrumentation and biopsy of these areas prior to scanning would compromise the accuracy of the subsequent radiological assessments. In addition, imaging may identify a potential primary site for a targeted biopsy.

Under general anaesthesia, each of the subsites of the head and neck should be examined under direct vision and by use of all types of straight and angled telescopes appropriate to that area. The subsites which should be examined are the nose, paranasal sinuses, nasopharynx, oral cavity, hard and soft palates, tongue base, tonsil, posterior pharyngeal wall, vallecula, supraglottis, glottis, subglottis, pyriform fossa, post-cricoid region and proximal oesophagus. Palpation of oral cavity and tongue base should also be carried out.

In any of these areas if there is any suspicion of ulceration, change in colour, asymmetry or fullness, then the area should be photographed and appropriate deep biopsies taken. If there is no obvious lesion, then the question of random biopsies arises. Although there is little evidence in support of this long-standing practice, biopsy of the post-nasal space, tongue base and/or pyriform fossa would still appear to be common practice especially if the positive lymph node is one of the first echelon lymph nodes draining the index site being biopsied.

There is an evolving evidence base in support of ever increasing oropharyngeal lymphoid tissue resection. It is now accepted that bilateral tonsillectomy should be carried out. An extension of this principle is an increasing body of evidence in support of excision or sampling the lingual tonsil (tongue base mucosectomy),^7^^–^^9^ which is best accomplished by transoral robotic surgery.^10^^,^^11^ Although this increases the yield of squamous carcinoma primaries the effect that this might have on structure and function within the oropharynx and ultimately how it relates to survival needs clarification.

Most current groups would suggest that PET–CT imaging, in conjunction with panendoscopy, directed biopsy as appropriate and bilateral tonsillectomy offer the greatest chance of identifying the occult primary tumour in the routine clinical setting. The role of tongue base mucosectomy by transoral laser or robotic approach, with or without PET–CT or HPV positivity needs prospective evaluation.

Following detailed clinical, radiological and operative assessment, if an index primary site is identified then treatment should be according to the guidelines for that site with nodal metastasis. If each of these investigations is negative, then this should be regarded as a ‘true’ unknown primary and the treatment considered as such.

### Staging

The neck is staged as set out elsewhere in this supplement. It should be noted that the correct T stage for an unknown primary is T0 and not TX.

## Treatment

The aim of the treatment of the majority of patients with a ‘true’ unknown primary tumour in the head and neck should be curative with the least morbidity to the upper aerodigestive tract possible. The treatment of an occult mucosal primary is often assumed and based on the well-studied natural history of mucosal squamous cell cancers of the upper aerodigestive tract. Most treatment regimens will therefore involve combined modality treatment, but on occasions, radiotherapy (RT), and even more rarely surgery, will be used as single modality treatment.^12^ The rate of emergence of the primary tumour is approximately 3 per cent per year, which is equivalent to the development of second carcinomas in the head and neck, lung and oesophagus. Therefore the primary aim of treatment is locoregional control. However, the rarity of unknown primaries (approximately 1–2 per cent of all squamous head and neck cancers) means there is a dearth of literature to guide best practice. Many of the management decisions are therefore controversial, and based on individual centre case series.

**Recommendations**

• All patients presenting with confirmed cervical lymph node metastatic SCC and no apparent primary site should undergo:
  • Positron emission tomography–computed tomography whole-body scan (R)
  • Panendoscopy and directed biopsies (R)
  • Bilateral tonsillectomy (R)
• Tongue base mucosectomy can be offered if facilities and expertise exist (G)

Surgery on its own may be sufficient treatment for N1 necks demonstrating no extracapsular spread, but in all other scenarios, needs to be supplemented by adjuvant (chemo) radiation (Table II).

For more advanced neck disease intensive combined treatment is required. This could be either a combination of neck dissection and RT or initial (chemo)-radiotherapy followed by planned neck dissection if a complete response is not evident on imaging. Both of these approaches appear to be equally effective. Of emerging significance is the question of HPV 16 and 18 positivity and the effect it has on treatment recommendations. Given the apparent good clinical response to HPV-positive lymph nodes then the question arises as to the advisability of surgical clearance of the neck with or without adjuvant (chemo) radiotherapy or whether primary RT should be considered as the only treatment modality in this specific group.

## Surgery

### T0N1

#### T0N1 – no extracapsular spread

Patients presenting with N1 disease and who are subsequently confirmed following surgery as having pN1 disease without extracapsular spread may be treated with surgery alone provided the surgery has been comprehensive. This should be in the form of a modified radical neck dissection (MRND), including levels 1–5, and in the vast majority preserving the ipsilateral sternomastoid muscle, internal jugular vein and accessory nerve. This has been shown to be as effective as RT and clearly avoids the potential side effects of RT. There are no randomised data to support MRND over selective neck dissection (SND).^13^ However, in the absence of other adjunctive therapies for the N1 neck, a MRND may be preferred as its extent and subsequent radiological assessment may avoid the need for radiation.

#### T0N1 – with extracapsular spread

When extracapsular spread is found, however, then RT to at least the involved nodal levels is necessary, although it is more usual to irradiate the entire ipsilateral post-operative neck, and boost the involved levels. The addition of chemotherapy to RT for occult primary head and neck cancer has not yet been established. However, as post-operative chemoradiation has been demonstrated to be superior to post-operative radiation alone in the context of pathologically confirmed extracapsular spread, in patients with detectable upper aerodigestive tract cancers, the addition of concomitant platinum-based chemotherapy to radiation should be considered.^14^ There are no robust data to support the additional use of total mucosal irradiation (TMI) with ipsilateral neck radiation following neck dissection for T0pN1 disease.

There are also some reports that locoregional tumour control is up to 40 per cent higher with surgery and radiation therapy compared with radiation alone, meaning radiation alone, even for N1 disease, must remain an option only for those who are inoperable on medical grounds or where it is considered appropriate for those who are HPV positive.

### T0N2a, T0N2b and T0N2c

For each of these stages comprehensive clearance of the involved lymph node levels is usually required in the form of MRND or SND with possible contralateral SND or MRND. The rate of regional recurrence for SND is similar to reported rates for MRND, when combined with adjuvant radiation, such that SND may be an appropriate surgical option for more advanced neck disease in selected patients. Equally in less advanced disease it has been reported that SND can be used with similar efficacy to MRND. Radical RT to one or both sides of the neck should be considered, even for pN2a disease, as in one of the largest series of occult primary head and neck cancer in 136 patients from the MD Anderson Centre, combined surgery and post-operative radiation was associated with lower rates of locoregional relapse and higher disease-free survival. This radiation may be given with or without concomitant chemotherapy as described above. While there remains no randomised data to support the use of chemotherapy for pN2 disease from an occult head and neck primary, there are two case series both demonstrating excellent progression-free survival (PFS) and overall survival (OS) rates. The chemotherapy protocols used were heterogeneous, and included concomitant cisplatin, concomitant 5-fluorouracil (5-FU) and hydroxyurea, as well as paclitaxel.

In the absence of supportive data, radiation of potential index sites, depending on the lymph nodes levels involved, remains controversial. It should remain an area of active investigation, with the conventional management of patients with pN2 disease being as described above.

### T0N3

It may not be possible to have a curative aim in patients with this staging. There is, however, a potential role for surgery as palliation, in the form of a radical neck dissection with the aim of preventing or delaying, the onset of fungation of the nodal metastasis. For curative intent a radical neck dissection or Type I MRND with post-operative chemoradiotherapy will usually be necessary.

**Table II:** Treatment recommendations

Stage	Surgery	Radiotherapy	Chemotherapy
T0N1 (no ECS)	SND or MRND	No unless for mucosal sites	No
T0N1 (ECS)	SND or MRND	Yes – either involved lymph nodes or ipsilateral neck and boost to involved lymph nodes	Should be considered
T0N2a, N2b, N2c	SND or MRND±contralateral SND or MRND	Yes – ipsilateral but bilateral should be considered	Should be considered
T0N3	Radical or type I MRND	Yes – ipsilateral but bilateral should be considered	Should be considered

SND = selective neck dissection; MRND = modified radical neck dissection

### Radiotherapy

#### Primary treatment

For N1 disease with extracapsular spread, N2 and N3 disease, initial chemoradiation with planned neck dissection only for those patients not achieving a clinical or metabolic complete response on post-treatment imaging is a valid management strategy.^12^^,^^15^ The extent of the RT fields to be treated is controversial. In the absence of high-level evidence, the practice of radiation therapy in this setting includes involved field only or bilateral neck and TMI. The latter is practiced commonly in the UK.

#### Adjuvant treatment

There is a lack of consensus on the RT target volumes that should be treated after neck dissection.^16^ Treatment of the ipsilateral hemi-neck alone is of considerably lower toxicity and has been shown to achieve local control rates in the cervical nodes of 90 per cent with contralateral relapse rates as low as 4.7 per cent, provided treatment strategies are determined using PET–CT. However, total mucosal and bilateral neck irradiation of the head and neck region is a common practice with the aim of eradicating the primary and the microscopic neck disease.

With the addition of cisplatin to primary RT for the treatment of head and neck cancer, an absolute survival benefit of 6.5 per cent is seen at five years. Investigating concomitant chemoradiation in the post-operative setting, the Radiation Therapy Oncology Group (RTOG) demonstrated a 10 per cent improvement in locoregional control rate, and a 22 per cent risk reduction of disease recurrence and death at two years, while the European Organisation for Research and Treatment of Cancer (EORTC) group showed a 13 per cent improvement in locoregional control, 25 per cent risk reduction of disease progression, and 30 per cent risk reduction of death at five years.^14^^,^^17^ These findings were based on the concomitant use of cisplatin 100 mg/m^2^ on days 1, 22 and 43, which must therefore remain the gold standard.

#### Total mucosal irradiation

This remains a controversial issue. In the largest series to date, no patient developed a metachronous primary in the follow-up period, and so would have experienced only toxicity rather than benefit from TMI. Some groups have recommended bilateral neck and TMI for occult primary head and neck cancer patients, claiming improved local control, but no OS benefit. There is no conclusive evidence to support the routine use of TMI.

What is clear, however, is that with conventional RT techniques, TMI is given at the price of significant acute toxicity and chronic morbidity, mainly xerostomia with its associated complications and effects on quality of life. Intensity modulated radiation therapy (IMRT) enables delivery of different doses during TMI, thus potentially reducing treatment related toxicity. Four centres have reported their experience of using IMRT to deliver TMI for unknown primaries, with excellent two-year locoregional control (85–88 per cent) and OS (74–85 per cent). The MD Anderson group, however, has most recently reported the most mature data, with five-year actuarial locoregional control of 94 per cent and OS of 89 per cent.^18^ The TMI in all reports was well tolerated, and with significantly reduced xerostomia and mucositis. Due to the lack of randomised evidence, the post-operative RT volume treated should therefore be at the discretion of the treating clinician. If TMI is advocated the use of IMRT is recommended.^19^^,^^20^

Radiation dosage schedules:

• Post-operative neck: 60 Gy in 30 fractions or equivalent
• Post-operative neck with extracapsular spread: 64–66 Gy in 32–33 fractions or equivalent
• Gross macroscopic disease still present: 70 Gy in 30 fractions or equivalent
• Putative mucosal sites and the uninvolved neck: 50 Gy in 25 fractions or equivalent.

### Chemotherapy

In the absence of randomised data to support chemotherapy, either before, during or after radiation for occult primary head and neck cancer, the indications for chemotherapy with post-operative or radical RT should be as for treatment of patients with detectable head and neck SCCs. The chemotherapy regimen used is at the discretion of the treating clinician, but will usually be platinum-based, single-agent cisplatin or carboplatin or cetuximab in patients with suboptimal renal function.

**Recommendations**

• Concomitant chemotherapy with radiation should be considered in patients with an unknown primary (R)
• Concomitant chemotherapy with radiation should be offered to suitable patients in the post-operative setting, where indicated (R)
• Neo-adjuvant chemotherapy can be used in gross ‘unresectable’ disease (R)

#### Neo-adjuvant chemotherapy

While the meta-analysis of chemotherapy in head and neck cancer (MACH-NC) failed to demonstrate a significant benefit for the use of induction chemotherapy,^21^ many of the historical trials included pre-dated the use of taxanes. Both the EORTC 24971 and TAX 323 studies and the TAX 324 trial found that the addition of docetaxel (T) to cisplatin (P) and 5-FU resulted in improved PFS, OS and response rate and yet lower associated toxicity. In the context of gross unresectable neck disease, it therefore seems reasonable to consider the use of such induction chemotherapy, particularly for patients with excellent performance status, as a cytoreductive measure prior to definitive concomitant chemoradiation, even for occult primary disease. The caveat remains that the outcome of such case series should be reported in the literature where possible, for this rare group.

#### Concomitant chemotherapy

The addition of post-operative adjuvant chemotherapy concurrently with radiation has transformed with the publication of two trials from EORTC and RTOG. See section ‘Adjuvant treatment’ for detailed discussion.

#### Adjuvant chemotherapy

There are no convincing data that chemotherapy given after radiation or surgery is of benefit in terms of either disease-free or OS for patients with detectable primaries. This approach cannot therefore be recommended for patients with occult primary head and neck cancer.

**Recommendations**

• Patients should be followed up at least two months in the first two years and three to six months in the subsequent years (G)
• Patients should be followed up to a minimum of five years with a prolonged follow-up for selected patients (G)
• Positron emission tomography–computed tomography scan at three to four months after treatment is a useful follow-up strategy for patients treated by chemoradiation therapy (R)

## Follow-up

Follow-up schedules should be in keeping with the monitoring of all patients who have received treatment for low-volume head and neck SCC with cervical metastasis, as discussed elsewhere in these guidelines. The highest risk period for relapse of squamous carcinoma following treatment occurs in the first two years. A frequent follow-up programme of monitoring every 4 weeks up to 18 months is indicated for patients who have received radical treatment. This should identify the appearance of a primary, or any recurrence, in turn allowing their prompt and optimal management.

As previously discussed, PET–CT is frequently a standard part of the work up for patients presenting with cervical metastasis from an occult primary. There are data to suggest that it also plays a useful role in follow-up. A negative PET–CT scan after treatment with chemoradiotherapy is associated with a high negative predictive value (>95 per cent), and a negative scan undertaken three to four months after completion of therapy can therefore provide some reassurance for the patient and clinician that there is no residual disease. However, there are no data on the value of subsequent imaging to monitor either subclinical locoregional recurrence or the development of a primary cancer, at a later stage. The decision regarding subsequent imaging, whether annually or otherwise, remains therefore at the discretion of the treating clinician.

### Key points

• All patients with a clinical unknown primary should have comprehensive imaging, including positron emission tomography–computed tomography imaging, followed by panendoscopy and bilateral tonsillectomy
• In the majority of cases, radical treatment should include surgical clearance of the neck followed by chemoradiotherapy
• Primary concurrent chemoradiation with planned neck dissection or neck salvage based on response is a valid alternative treatment strategy
• If total mucosal irradiation is to be considered, then intensity modulated radiation therapy should be used
• Follow-up should be similar to that employed in patients who have received the treatment for an identified tumour of the head and neck.
